# Targeted EGFR Nanotherapy in Non-Small Cell Lung Cancer

**DOI:** 10.3390/jfb14090466

**Published:** 2023-09-09

**Authors:** Andreea Crintea, Anne-Marie Constantin, Alexandru C. Motofelea, Carmen-Bianca Crivii, Maria A. Velescu, Răzvan L. Coșeriu, Tamás Ilyés, Alexandra M. Crăciun, Ciprian N. Silaghi

**Affiliations:** 1 Department of Molecular Sciences, University of Medicine and Pharmacy “Iuliu Hațieganu”, 400349 Cluj-Napoca, Romania; crintea.andreea@umfcluj.ro (A.C.); tamas.ilyes@umfcluj.ro (T.I.); silaghi.ciprian@umfcluj.ro (C.N.S.); 2 Department of Morphological Sciences, University of Medicine and Pharmacy “Iuliu Hațieganu”, 400349 Cluj-Napoca, Romania; annemarie.chindris@umfcluj.ro (A.-M.C.); bianca.crivii@umfcluj.ro (C.-B.C.); 3 Department of Internal Medicine, University of Medicine and Pharmacy “Victor Babeș”, 300041 Timișoara, Romania; alexandru.motofelea@umft.ro; 4 Faculty of Medicine, University of Medicine and Pharmacy “Iuliu Hațieganu”, 400349 Cluj-Napoca, Romania; velescu.mariaamalia@elearn.umfcluj.ro; 5 Department of Microbiology, University of Medicine, Pharmacy, Science and Technology “George Emil Palade”, 540142 Târgu-Mureș, Romania; lucian-razvan.coseriu@umfst.ro

**Keywords:** lung cancer, EGFR, non-small cell lung cancer, nanotherapy

## Abstract

Non-small cell lung cancer (NSCLC) remains a leading cause of cancer-related mortality worldwide. Despite advances in treatment, the prognosis remains poor, highlighting the need for novel therapeutic strategies. The present review explores the potential of targeted epidermal growth factor receptor (EGFR) nanotherapy as an alternative treatment for NSCLC, showing that EGFR-targeted nanoparticles are efficiently taken up by NSCLC cells, leading to a significant reduction in tumor growth in mouse models. Consequently, we suggest that targeted EGFR nanotherapy could be an innovative treatment strategy for NSCLC; however, further studies are needed to optimize the nanoparticles and evaluate their safety and efficacy in clinical settings and human trials.

## 1. Lung Cancer Awareness

Lung cancer, the leading cause of cancer-related mortality worldwide, continues to show alarming incidence rates and poses complex problems for the medical community [1].

According to the Lung Cancer Research Foundation, smoking is the leading cause of lung neoplasm in smokers, this condition alone commonly accounts for all lung cancer patients. Furthermore, the remaining individuals may have been exposed to radon, other air pollutants, secondhand smoke, or other common teratogenic agents [2].

According to the GLOBOCAN Lung Cancer Facts Sheet and the National Health Service (NHS), lung cancer is strongly age-dependent, predominantly affecting individuals over the age of 40 and especially those over the age of 75 [3,4,5]. Looking at the sex ratio incidence and death worldwide, with few exceptions, males appear to be significantly more affected, although a recent study found that this incidence paradigm is completely reversed in young women and men due to identical smoking practices [6,7].

Detecting lung cancer at a very early stage remains difficult as significant symptoms or signs do not manifest until the tumor has progressed. According to the National Cancer Institute (NCI), only 18% of early-stage lung cancer cases in the US are detected when the tumor is still localized, while 56% are detected after the cancer has spread to other parts of the body [8,9,10]. However, the gratifying news is that, according to the same report, both lung cancer incidence and mortality have been in a slight but constant decline from 1992 to 2019 in the USA, which may be attributed to new and enhanced diagnostic tools, such as circulating microRNAs detection, ctDNA, or specific antibody identification [8,11,12,13]. This general tendency to move the focus from basic diagnosis and prognosis instruments, such as X-ray, CT, or low-dose CT (LDCT) screenings, to molecular approaches outlines the importance and relevance of the tumor microenvironment as well as targeted nano-therapy [14,15,16].

Based on the prognosis and treatment patterns, there are two main types of lung cancer: non-small cell lung cancer (NSCLC) and small cell lung cancer (SCLC). NSCLC accounts for approximately 80–85% of all lung cancer cases, while SCLC occurs in the remaining approximately 15–20% of cases, as reported by most governmental cancer surveillance agencies worldwide. Another sub-division of NCSLC includes adenocarcinomas (ADs), squamous cell carcinomas (SCCs), and large cell carcinomas [17].

ADs account for approximately 40–50% of lung cancer cases. These begin in mucus-producing cells and are strongly associated with smoking habits [18,19].

SCCs account for about 20 to 30% of all lung carcinomas and are strongly associated with tobacco smoke as the primary causal agent [20]. It develops due to the transformation of the squamous epithelial cells that border the airways [21].

Large cell carcinomas, accounting for only 10 to 20% of lung malignancies, are distinguished by the tumor’s quick growth and dissemination and are easily spotted on routine chest radiographs because of the bulky large mass visible on the chest images [22,23]. 

SCLC, named for the microscopic appearance of the tumor, presents more challenges to diagnose than NSCLC. According to the American Cancer Society, this is mainly because of its rapid growth and spread. However, chemo- and radiotherapy have shown excellent results in treating this type of lung cancer [24,25].

Finally, several uncommon types of lung cancer require special treatment procedures, such as lymphomas, mesotheliomas, adenosquamous carcinoma, or large cell neuroendocrine carcinoma [26,27,28].

**Figure 1:**
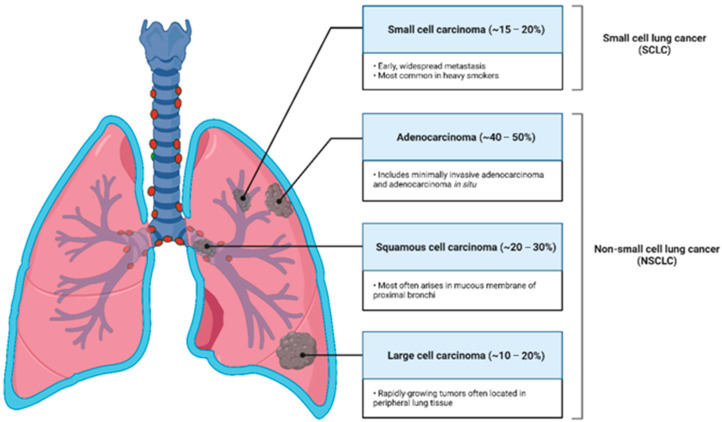
Lung cancer classification, according to terminology for small biopsies, cytology specimens, and resection specimens. Small cell carcinoma; Adenocarcinoma; Squamous cell carcinoma; Large cell carcinoma.

With a habitual occurrence in older people, the current paradigm of pulmonary neoplasms might be drastically changed for several reasons. Considering current social and economic tendencies, with many emergent and youth-appealing alternatives [24,25] for tobacco use being developed in the latest years and being embraced by (but not limited to) the young portion of the population, lung cancer remains a concerning and relevant topic in the future [29]. The types of lung cancers are summarized in Figure 1.

## 2. Epidermal Growth Factor Receptor in Pulmonary Pathology

The epidermal growth factor receptor (EGFR) is an essential proto-oncogene in the context of NSCLC trigger and growth. Part of the receptor tyrosine kinases (RTKs) family, this protein binds ligands such as epidermal growth factor (EGF), transforming growth factor alpha (TGF-α), betacellulin (BTC), amphiregulin (AREG), or epigen (EPGN), transforming the linkage of these molecules to intra-cytoplasmic signaling cascades. Thus, EGFR converts the binding of extracellular ligands into corresponding intracellular responses [30,30,31,32,33,34,35].

The extracellular portion of the EGFR is the most significant portion, comprising 621 amino acid residues. It also has a helical, transmembrane region consisting of only 23 amino acids and a cytoplasmicregion, which is composed of 542 amino acids [36,37]. The receptor’s extracellular domain will undergo homo- or heterodimerization upon ligand binding; particular cytoplasmic residues will further undergo autophosphorylation [38].

**Figure 2:**
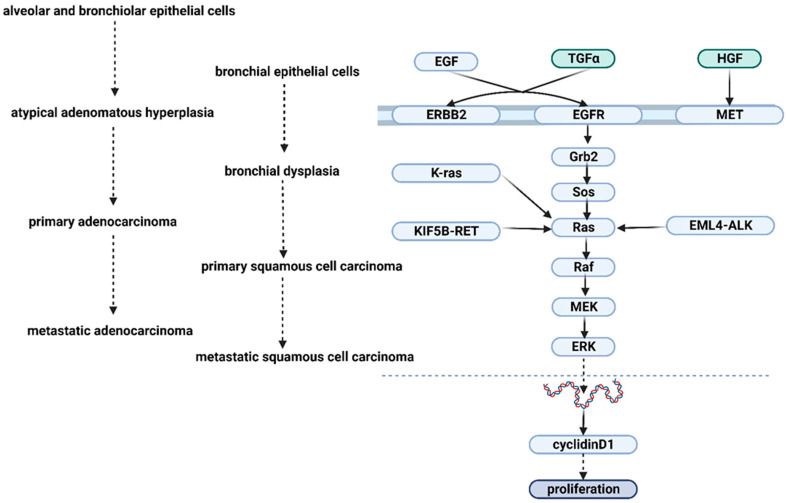
EGF signaling pathway in the proliferation of NSCLC. Abbreviations: EGF, epidermal growth factor; TGFα, Transforming growth factor alpha; HGF, Hepatocyte growth factor; ERBB2, tyrosine-protein kinase erbB-2 receptor; EGFR, epidermal growth factor receptor; *MET*, proto-oncogene tyrosine-protein kinase Met; Grb2, growth factor receptor-bound protein 2; SOS, son of sevenless; Ras, GTPase HRas; *Raf*, A-Raf proto-oncogene serine/threonine-protein kinase; MEK, mitogen-activated protein kinase 1; ERK, mitogen-activated protein kinase 1/3; cyclidinD1, G1/S-specific cyclin-D1; KIF5B-RET, kinesin family member 5; EML4-ALK, anaplastic lymphoma kinase; K-ras, GTPase KRas.

The primary intracellular signaling pathways will subsequently be activated by this active phosphorylated form of the receptor, which will also attract adaptor proteins like son of sevenless (SOS), growth factor receptor-bound protein 2 (GRB2), GTPase HRas (RAS), A-Raf proto-oncogene serine/threonine-protein kinase (RAF), or others [30,39,40]. EGFR not only plays a crucial role in processes such as angiogenesis and the suppression of apoptosis but also activates and modifies important cell pathways (Figure 2) [30,41,42,43].

In addition, Shostak and Chariot [44] hypothesized that the relationship between EGFR and nuclear factor kappa B (NF-κB), another intracellular complex signaling pathway, regulates the growth of solid tumors and is common in analyzed cancerous tissues.

The importance of EGFR in pathological contexts might explain the abundance of literature resources discussing the involvement of EGFR in various biological processes. The undeniable role of this transmembrane protein in the pathogenesis of lung cancer underscores the significance of EGFR not only for the current review but for lung cancer research in general, even though the range of EGFR-related health issues is not very broad. In addition, a study found that mutations in the gene that codes for EGFR can cause inflammatory skin and bowel disease in newborns as well as identifying its relevance in the setting of pulmonary etiology [45].

In the UniProt database alone, there are more than 30 potential EGFR mutations that have been linked to the development of lung cancer, distinguishing two primary types of mutations that could lead to pro-tumorigenic activity: alterations to the extracellular domain and accidental changes to the kinase domain. 

The first category, in which EGFR has a truncated extracellular domain, implies the formation of a so-called epidermal growth factor receptor variant III (EGFR vIII, considered the most common and most notable mutation [46,47,48]). A junction between exons 1 and 8 and a novel glycine residue are both created at this junction site when 801 base pairs coding 267 amino acid residues are deleted from the *EGFR* gene, shortening the N-terminus of this protein, which encodes the extracellular region of the receptor [49,50]. The resulting mutant protein was described as constitutively active, despite having poor signaling activity and being unable to bind any recognized ligand [46]. We can easily anticipate the unfortunate involvement of this mutant variant of the receptor in human cancers given the significance of EGFR signaling in normal cell proliferation and differentiation. Additionally, it was demonstrated that EGFR vIII may co-express with the regular EGFR and might indirectly influence its activity by inducing the production of its ligands, heparin-binding EGF and TGF-α [51,52]. Furthermore, Pillay et al. [53] demonstrated that this mutant protein also activates additional receptor tyrosine kinases (RTKs), such as hepatocyte growth factor receptor (HGFR), VEGF receptor 2 (VEGFR2), or platelet-derived growth factor receptor (PDGFR), which are crucial in the regulation of the cell cycle. Finally, EGFR vIII was also linked to enhanced anaerobic glycolysis and lipogenesis processes, via the phosphatidylino-sitol-4,5-bisphosphate-3-kinase catalytic subunit alpha/beta/delta/RAC serine/threonine-protein kinase/mechanistic target of the rapamycin (PI3KCA/AKT/mTOR) pathway, a fact that strengthens its position as a pro-tumoral mutated form of EGFR [54,55,56,57].

Even if the presence of EGFR vIII has been more intensely studied in the context of brain cancers, studies have highlighted its emergence in pulmonary neoplasms. However, there are slightly conflicting results, with several authors [58] stating that this mutated form was reported only in about 5% of human lung SCCs and never in ADs, while others [46] presented evidence for up to 41% presence in SCCs and up to 41% presence in ADs. These differences are mainly due to the different techniques used to assess EGFR vIII (from PCR to immunohistochemistry and Western blot) and technical limitations related to the poor availability and affinity of EGFR vIII antibodies for the respective protein [46,59].

Genomic changes in EGFR kinase domains, discovered after extracellular domain mutations, were shown to be much more significant and frequent in lung cancer formation, notably in NSCLC. These mutations, which comprise around 16% of NCSLCs, typically involve DNA alterations that cause replacements of amino acids [58,60].

**Figure 3:**
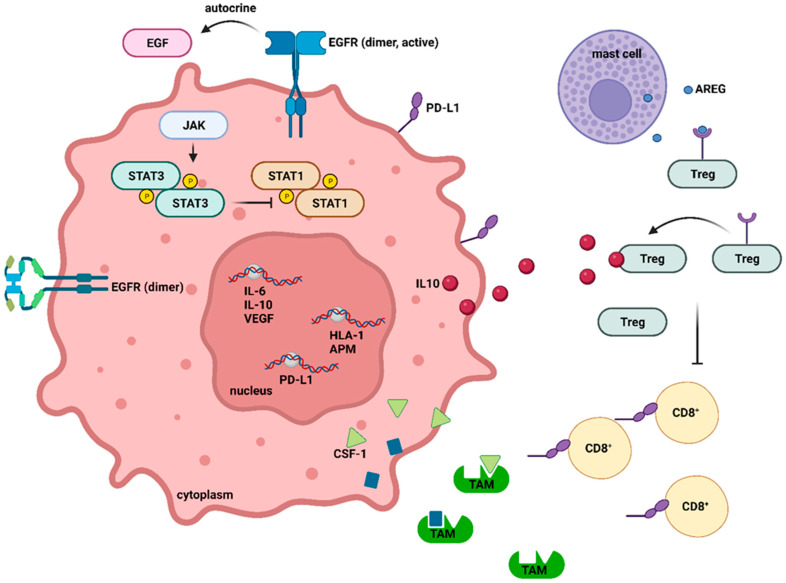
EGF signaling pathway and the tumor microenvironment (modified after [70]). Abbreviations: EGF, epidermal growth factor; EGFR, epidermal growth factor receptor, JAK, Janus kinase; STAT3, signal transducer and activator of transcription 3; STAT 1, signal transducer and activator of transcription 1; IL-6, interleukin 6; IL-10, interleukin 10; VEGF, vascular endothelial growth factor; HLA-1, human leukocyte antigen 1; APM, antigen processing machinery; PD-L1, programmed death-1 ligand 1; CSF-1, colony-stimulating factor 1; TAM, Tyro3, AXL, MerTK; CD^8+^, cluster of differentiation 8; Treg, regulatory T cells; AREG, amphiregulin.

One of the most well-known genetic variations in this group is G719S, in which serine takes the place of a glycine residue at position 719. Briefly, mutations in this domain cause abnormal increased autophosphorylation episodes that artificially activate the EGFR and cause the receptor to initiate signaling pathways through signal transducers within the tumor microenvironment (Figure 3). Some of these uncontrolled signaling cascades may result in pro-tumoral phenotypes [61].

While the G719S mutation is not frequently observed in lung cancers (only 0.22% of NSCLC cases had this mutation), its clinical significance makes it worth mentioning [62]. Lynch et al. [63] demonstrated that screening for RTK mutations in lung cancer patients might establish effective therapy strategies from the very early stages of medication and/or indicate the recommended drug substitute in cases of drug-resistance development. Their study identified identical G719S somatic mutations in multiple Gefitinib-responsive patients with NSCLC and proved that individuals without similar mutations were also unresponsive to gefitinib.

On a molecular level, it is thought that the G719S mutation controls the interaction between the receptor and this ligand by repositioning critical ATP-binding residues of EGFR. This occurs primarily because ATP and gefitinib compete with one another to bind to the EGFR, with ATP acting as an activator and gefitinib as an inhibitor of receptor activity. Thus, by screening for these changes in patients, physicians could quickly determine the best course of action, considerably increasing the likelihood that lung cancer patients will survive. This mechanism and others have also paved the way for developing specialized and targeted therapies for treating patients with malignancies harboring EGFR mutations [64,65,66].

Last but not least, despite the potential of these screenings being foreseen around 20 years ago, technical barriers severely limited their use on a large scale. Novel techniques, such as liquid biopsy and digital multiplex PCR, were shown to promote and enhance the therapeutic value of EGFR mutation screenings even more. The most important advantages of these strategies are greatly improved sensitivity and accuracy especially when ascertaining therapy approaches for patients with advanced stages of NSCLC [67,68]. Furthermore, these recently established methods may be beneficial for patients with insufficient solid tissue biopsies or those with poor-quality tumoral samples [69], as well as for screening several EGFR mutations in a single assay while utilizing small amounts of plasma [67].

As a result of its value as a diagnostic and therapeutic tool, EGFR plays a significant role in the management of lung cancer. After over three decades of being acknowledged as a crucial molecule in pulmonary pathology, cutting-edge approaches have shown new ways that EGFR may function as a marker and a component in determining treatment, both in the advanced and early stages of NSCLC.

Further on, we will circumscribe the most relevant aspects related to targeted EGFR nanotherapy in NSCLC patients.

## 3. Therapeutic Management of NSCLC

According to the American Cancer Society, there will be at least 238,340 new cases of lung cancer and around 127,070 lung cancer deaths in the United States in 2023 [71]. Among lung cancer cases, the 5-year relative survival rate of patients with NSCLC is 64% for the localized stage, 37% for the regional stage, 8% for those with distant metastases, and 26% for overall survival [72].

**Figure 4:**
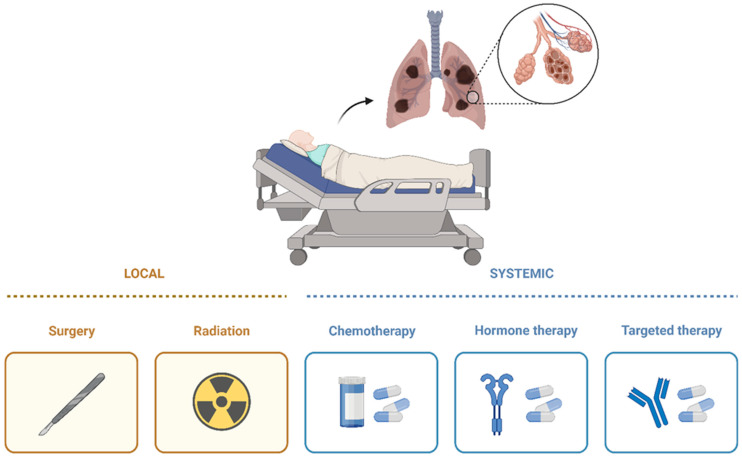
Therapeutic management options for NSCLC.

The treatment options for these patients vary, depending on the type and stage of cancer and the possible side effects. There are systemic and local therapies, the main classes being chemotherapy (ChT), targeted therapy, immunotherapy, radiotherapy (RT), and surgery [73] (Figure 4).

### 3.1. Management of Early-Stage NSCLC

Stage 0 NSCLC is limited to the lining layer of the airways. It may be AD in situ (AIS) or SCS in situ (SCIS). An NSCLC tumor stage I (A, B) is 4 cm or less in size and has not spread to any lymph nodes. A stage II (A, B) tumor is 5 cm or more in diameter and does or does not involve the lymph nodes within the lung (N1) [74,75,76]. Usually, these stages can be treated with surgery alone. 

For stage 0, the recommended surgery is segmentectomy or wedge resection (removal of part of the lobe). Alternative treatments are photodynamic therapy (PDT), laser therapy, or brachytherapy [38,41,77].

For Stage I, surgery is still an option (lobectomy, sleeve resection, segmentectomy, or wedge resection). Adjuvant ChT or RT may be suggested in specific circumstances [78].

In the LACE collaborative group study, Pignon et al. [79] demonstrated a 5.4% increase in the absolute benefit five-year survival rate with adjuvant ChT (cisplatin). Immunotherapy with nivolumab and ChT before surgery is another option for treating tumors larger than 4 cm. Stereotactic body radiation therapy (SBRT) constitutes a possible alternative to surgery [41,77].

### 3.2. Management of Locally Advanced (Stage III) NSCLC

Stage III is divided into IIIA, IIIB, or IIIC according to the size of the tumor and the affected lymph nodes. Stage III tumors typically do not have distant metastases but cannot be treated surgically alone [74]. Resectable tumors include N2-type tumors without affecting other lymph nodes, T4N0, or those that can be removed surgically following induction therapy if nodal downstaging has occurred and a pneumonectomy can be avoided. Chemo-radio therapy (CRT) may be suggested before and after surgery [80].

Treatment options depend on the stage III subtypes and the decision of a multidisciplinary team.Stage IIIA treatment entails a combination of RT, ChT, and surgery. The treatment plan usually starts with neoadjuvant CRT. Alternative treatments include immunotherapy with nivolumab along with ChT first and then surgery; thus, surgery or immunotherapy (pembrolizumab and cemiplimab) as the first line of treatment. For patients with EGFR-mutated NSCLC, the targeted drug osimertinib could be used as an adjuvant treatment [80,81].

Stage IIIB cannot be removed entirely by surgery. If the condition remains stable for at least two months following CRT, the treatment consists of CRT followed by immunotherapy (Durvalumab). Other first-line therapies include RT, ChT, and immunotherapy with pembrolizumab or cemiplimab [80,81].

For Stage IIIC, therapy options include sequential or concurrent ChT and RT, RT alone, new fractionation schedules, radiosensitizers, combined-modality approaches, or targeted drug delivery in patients with EGFR-mutated or ALK-translocated cancers, and adaptive radiation therapy with response monitoring based on positron emission tomography (under clinical evaluation) [81,82,83,84,85]. 

### 3.3. Management of Late-Stage IV(A) and IV(B) NSCLC

The patient’s overall health, histology, molecular pathology, age, patient’s health status, comorbidities, location of distant metastases, genetic traits, and protein alterations in tumor cells all influence the treatment options [81,86]. As in the case of stage III, stage IV is also subdivided into IVA and IVB.

In the case of stage IVA, one remote location (usually the central nervous system) is affected. Initially, surgery, stereotactic radiation, or surgery followed by RT to the entire brain are used to treat the metastases. The primary tumor may be treated with surgery, ChT, RT, or a combination of these [81,86,87].

**Figure 5:**
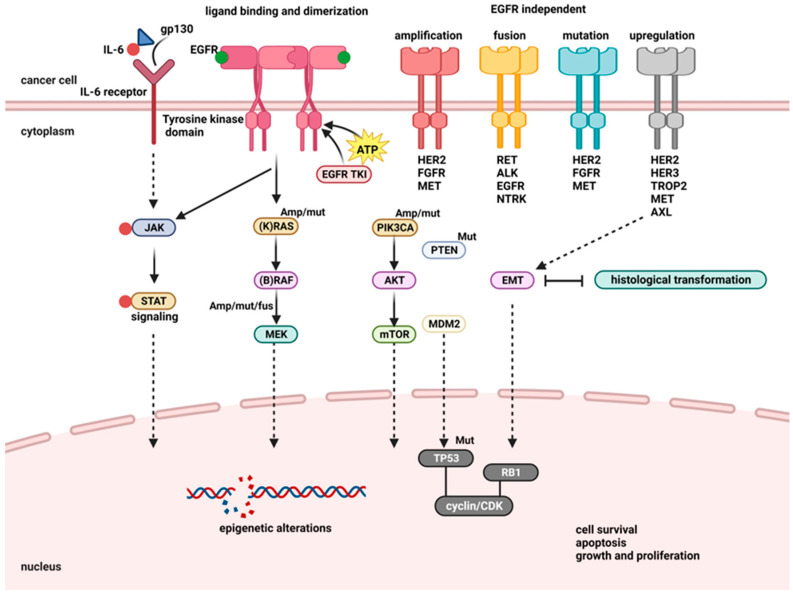
EGFR signal transduction pathway—an overview (modified after [94]). Abbreviations: gp130, glycoprotein 130; IL-6, interleukin-6; JAK, Janus kinase; STAT, signal transduction and activator of transcription; EGFR, epidermal growth factor receptor; EGFR-TKI, epidermal growth factor receptor tyrosine kinase inhibitor; (K)RAS, GTPase KRas; (B)RAF, B-Raf proto-oncogene serine/threonine-protein kinase; MEK, mitogen-activated protein kinase kinase 1; PIK3CA, phosphatidylinositol-4,5-bisphosphate 3-kinase catalytic subunit alpha/beta/delta; AKT, RAC serine/threonine-protein kinase; mTOR, mechanistic target of rapamycin; PTEN, phosphatase and tensin homolog; MDM2, mouse double minute-2 homolog; TP53, tumor protein 53; EMT, epithelial–mesenchymal transition; RB1, epithelial–mesenchymal transition; HER2, human epidermal growth factor receptor 2; FGFR, fibroblast growth factor receptor; *MET*, proto-oncogene tyrosine-protein kinase Met; *RET*, proto-oncogene tyrosine-protein kinase Ret; ALK, anaplastic lymphoma kinase; NTRK, neurotrophic tyrosine receptor kinase; HER3, human epidermal growth factor receptor 3; TROP2, trophoblast cell-surface antigen 2.

In stage IVB, two or more remote sites are involved. Prior to developing a treatment plan, gene mutations in the *KRAS, EGFR, ALK, ROS1, BRAF, RET, MET, or NTRK* genes involved in the EGFR signal transduction pathway should be assessed (Figure 5) with specific therapies to be considered (e.g., sotorasib—*KRAS* G12C gene mutation, crizotinib—*ROS1* gene mutation, etc.). Immunotherapy medications (immune checkpoint inhibitors), either alone or in combination with ChT, are advised as additional treatments. If there are no hemorrhagic risks, bevacizumab and ChT may be used to treat SCC patients [81,86,88].

### 3.4. Management of Recurrent NSCLC and Palliative Care

If there is a relapse of NSCLC, the type of treatment used will be dictated by the location and extent of the tumor, the treatments used beforehand, and the patient’s health. The second line of treatment is represented by ChT (docetaxel and pemetrexed), RT, or targeted therapy. Certain types of NSCLC can be treated with an immunotherapy drug such as nivolumab, along with ipilimumab, pembrolizumab, or atezolizumab [89,90,91].

For patients needing palliative care, RT may be utilized to treat tracheal, esophageal, or bronchial compression, pain, vocal cord paralysis, hemoptysis, and superior vena cava syndrome. Proximal obstructive lesions have also been treated with endobronchial laser therapy and brachytherapy. There are no differences regarding the efficacy of either form of RT. However, there is evidence that patients with better performance status who receive larger doses of radiation do live slightly longer (5% at one year and 3% at two years) [92,93].

Regardless of the cancer stage, the tumor cells can present various resistance mechanisms to the treatments administered, considerably reducing the effectiveness of treatment.

As seen in Figure 5, EGFR is a protein expressed on the surface of cells, involved in cell growth and division. The phosphorylation of EGFR is caused by ligand-induced dimerization of the receptor monomer, which brings intracellular kinase domains into proximity to one another for trans-autophosphorylation, which activates downstream signaling cascades. In cancer, inappropriate EGFR activation is triggered by amplification, point mutations, transcriptional upregulation, and ligand overproduction induced via autocrine/paracrine pathways. NSCLC cells contain an excess of EGFR, which causes them to grow quicker, and inhibitors of EGFR can block the EGFR signal, leading to cell proliferation [95].

## 4. Mutations-Related EGFR Treatment and Targeted Nanotherapy in NSLC

**Figure 6:**
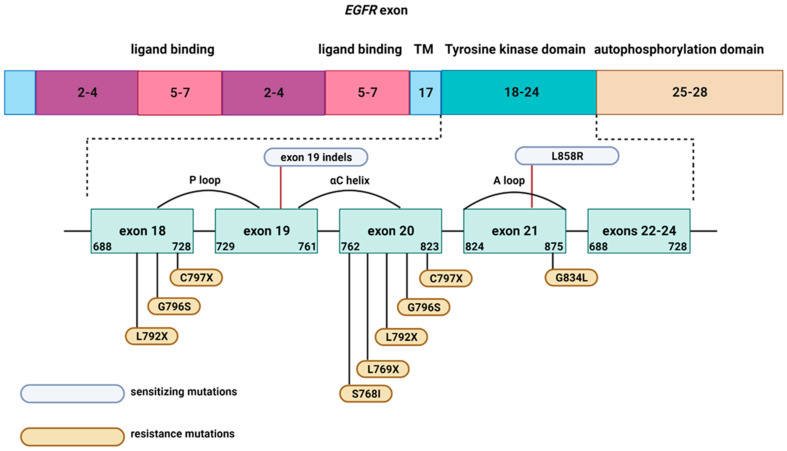
Sensitizing or resistance mutations in EGFR (modified after [94]). Abbreviation: TM, trans-membrane domain.

In recent years, the focus on EGFR mutations in NSCLC has acquired significant therapeutic implications. In addition to the presence of specific clinical features, such as non-smoker, Asian ethnicity, female gender, and AD histology, the patients with EGFR activating mutations (e.g., exon 19 deletions or exon 21 L858R point mutation) have access to specific treatments (EGFR-TKI—e.g., gefitinib), leading to a significantly higher overall survival [96]. There are four generations of EGFR-TKIs for NSCLC, each targeting different EGFR mutations and binding reversibly or irreversibly to EGFR. With the presence of EGFR mutations comes the possibility of developing resistance/tolerance to the initial treatment via de novo EGFR mutations and the need for substitution with ChT or other therapies. The known mechanisms involved in the development of resistance to EGFR-TKI are primary (no response to initial treatment) or acquired (initial response to therapy, then resistance) mutations (Table 1) [97,98]. Each generation of EGFR-TKIs has specific sensitizing mutations, lowering treatment efficiency. Del19 and L858R mutations are involved in reduced response to the first generation of EGFR-TKIs whereas Del19, L858R, and T790M mutations are incriminated for the second EGFR-TKIs generation. The third and fourth generations have decreased effectiveness when mutations, such as Del19, L858R, T790M, and C797S are present [97,99,100]. Osimertinib, a third-generation EGFR-TKI, is an effective first- and second-line NSCLC therapy but patients eventually develop resistance to the drug, thus requiring alternative treatment options [101]. The sensitizing and resistance mutations in EGFR are summarized in Figure 6.

Various studies have been conducted to identify potential solutions to resistance in NSCLC EGFR-mutated tumors (sensitizing, primary, or acquired mutations). Yi et al. [102] concluded, in a meta-analysis that included 10 studies with a total of 1528 patients from January 2023, that combining gefitinib with ChT would have significant improvements in the objective response rate (ORR) (OR = 1.54; 95% CI 1.13–2.1; *p* = 0.006), disease control rate (DCR) (OR = 1.62; 95% CI 1.14–2.29; *p* = 0.007), progression-free survival (PFS) (HR = 1.67; 95% CI 1.45–1.94; *p* < 0.001), and overall survival (OS) (HR = 1.49; 95% CI 1.2–1.87; *p* < 0.001) compared to gefitinib alone. The only downside was represented by the higher incidence of complications, mainly due to the ChT [OR of 3.29, 95% CI 2.57–4.21; *p* < 0.001)]. One of the possible explanations was the intratumoral genetic heterogeneity for targets such as EGFR, which could be prevented via the usage of ChT followed by an EGFR-TKI treatment, lowering the risk of EGFR-sensitizing mutations. 

A study realized by Rocco et al. [103] underlines the higher PFS but the lack of improvement in the OS when combining antiangiogenic monoclonal antibodies with EGFR-TKIs in the treatment of NSCLC with EGFR-mutations. Another study, conducted by Wang et al. [104] in 2022, focused on the tight relationship between EGFR and vascular endothelial growth factor A (VEGF), as both play an essential role in tumoral growth. The study tried to prove the positive outcome of dual-inhibition of EGFR-VEGF, the main results being the resistance to acquired mutations of tumor cells to EGFR inhibitors and increasing PFS times, but the disadvantage of this treatment would be the adverse reactions (e.g., renal dysfunction) [105].

Another meta-analysis (Dai et al. [106]), focused on the overall benefits of using ChT plus EGFR-TKIs compared to antiangiogenic agents with EGFR-TKIs in advanced EGFR-mutated NSCLC proved only the higher ORR in the first arm (RR = 1.1, 95% CI: 1.0–1.2), without any significant differences in PFS, OS or DCR. Del19 and L858 mutations of exon 21 led to similar survival benefits in the two arms.

**Table 1:** Mechanisms of resistance to EGFR-TKIs.

Mechanisms of primary resistance	References
Exon 20 insertions	[107]
T790M mutation	[46,108]
HGF overexpression	[108]
BCL2L11 deletion	[46,109]
**Mechanisms of acquired resistance**	**References**
T790M gatekeeper mutation in the ATP binding pocket of EGFR	[109,110]
D761Y, L747S and T854A mutations	[48]
*MET* gene amplification	[108,110]
PI3KCA mutation	[110]
Histological transformation	[111,112]
HGF overexpression	[113,114]
IGF-1R hyperphosphorylation	[114]
C797S mutation	[49]
G796R/S/, l792H, L718Q, and G724S substitutions	[110,115]

Abbreviations: BCL2L11, Bcl-2 interacting mediator of cell death; IGF-1R, insulin-like growth factor-1 receptor.

Nanoparticles (NPs) have gained popularity in the last decades, representing the next generation of treatments. According to the Food and Drug Administration (FDA), NPs are “materials that have at least one dimension in the range of approximately 1 to 100 nm and exhibit dimension-dependent phenomena” [116]. Nanomaterials are used in the medical domain for drug and gene delivery, bio-detection of pathogens, probing of DNA structure, tumor destruction via heating (hyperthermia), and many other purposes such as integration of anticancer chemo/immuno-based drugs with multifunctional nanomedicines that have an imaging modality to determine tumor location and can respond to stimuli such as light, pH, magnetic field, or metabolic changes to trigger ChT, photothermal therapy, gene transfection, photodynamic therapy, radiotherapy, or catalytic therapy to increase tumor immunogenicity [117,118,119]. 

## 5. Nano-Immunotherapies for EGFR Mutated NSCLC

NPs are an attractive delivery system because the drugs are protected from degradation while circulating through the body and the organism is simultaneously protected from drug-related toxicity [115]. There was initial optimism surrounding the passive targeting of NPs to tumors via the enhanced permeability and retention (EPR) effect, based on the increased permeability of leaky blood vessels around tumors enabling the accumulation of NPs at the desired site.

Despite credible theoretical underpinnings, the EPR effect proved to have limited efficacy and low reliability in clinical practice and is generally insufficient for the targeting of NPs to tumor sites [120]. Furthermore, once at the tumor site, NPs would benefit from an active tumor-targeting system based on cancer cell surface markers. For EGFR-expressing cancer cells, as in the case of NSCLC, a host of EGFR-binding ligands were developed to be conjugated onto NPs. These were reviewed in detail by Nguyen et al. [121] and include peptides like EGF, the EGFR-binding peptide GE11, and anti-EGFR antibodies, or antibody-like molecules, such as monoclonal antibodies (mAb), fragment antigen-binding (Fab) regions, single-chain fragment variable (ScFv) antibodies, nanobodies, and aptamers [121].

Coupling the targeting ability of antibodies with the drug delivery capabilities of NPs holds great promise for the treatment of various cancers, therefore prominent examples of EGFR-targeting NPs are detailed in the following sections.

## 6. Nanoparticles Suitable for NSCLC

**Figure 7:**
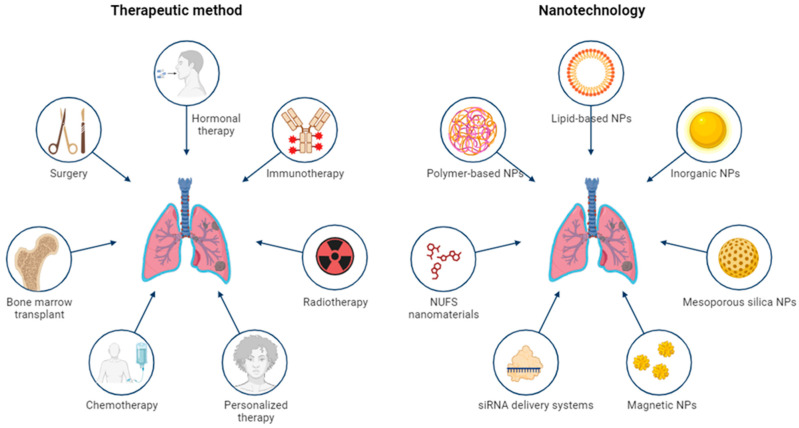
Nanotechnology and therapeutic methods used in NSCLC.

There are numerous types of NPs in use or in studies, such as lipid-based, polymer-based and micelles, dendrimers, carbon-based, metallic, magnetic, etc. [122]. The comparison between therapeutic methods and nanotechnology used in NSCLC is summarized in Figure 7.

Regarding NSCLC, numerous ongoing studies involving lipid NPs-based therapies focus on delivering the active drug or gene therapy to the tumor site and reducing the side effects. Some of these studies are clinical trials based on lipid NPs and mRNA in different phases, from I to IV. [5] Some examples are BIND-014 (docetaxel NPs) [6], paclitaxel albumin-stabilized NPs [7], CRLX101 (cyclodextrin-based polymer with camptothecin) [8], and GPX-001 (TUSC2 encapsulate in lipid NPs) [9].

### 6.1. Organic Nanomaterials

#### 6.1.1. Polymer-Based Particles

Synthetic polymers, such as polylactic acid, polyglycolic acid, and polyhydroxy butyrate, are usually suitable for drug delivery due to their individual properties, such as biocompatibility and biodegradability [116]. One of the first studies on NPs was published in 2011 and focused on the usage of EGFR-targeted heparin-cis-diamminedichloroplatinum (II) (EHDDP) NPs for the delivery of ChT to the tumor site in NSCLC. Some of the main advantages of this NP would be that heparin is biocompatible and biodegradable and that the chemically modified variant did not show any anticoagulant activity. The targeted delivery of DDP by EHDDP NPs significantly reduced the associated toxicity to the kidney and spleen due to a higher DDP concentration at tumor sites and a longer time to renal excretion, which was demonstrated both in vitro and in vivo studies [123].

Gemcitabine (2’,2’-difluoro 2’-deoxycytidine, GEM) is an analog of cytosine arabinoside (Ara-C) and is frequently used as a first line of therapy in SCS and in unfit elderly patients [124]. Gemcitabine-loaded cetuximab (CET) surface-modified poly(lactic) acid (PLA) NPs (CET-GEM/PLA NPs) targeting EGFR overexpressing A549 NSCLC cells determined a two-fold increase in fluorescent intensity compared to that of non-targeted NPs in the cancer cells and a greater level of cell apoptosis (early and late apoptosis ∼40%) [125].

Combinatorial-designed EGFR-targeted chitosan (CS) NPs with lipid-modified platinum derivatives (cisplatin) were tested for their encapsulation efficiency and in vitro cytotoxicity. They were more effective in suppressing cellular growth in both sensitive and resistant lung cancer cells than the drug solution. The increase in potency can be a benefit to therapy and limit side effects by reducing the therapeutic dose. These vectors can be modified to load a variety of therapeutic agents with different hydrophobicity [126]. Another nano-drug delivery system for co-encapsulate hydrophilic (carboplatin) and hydrophobic anti-tumor drugs (paclitaxel) was created in 2016 to reduce the tumoral drug resistance and the influence on normal cells and tissues. Its efficiency was evaluated in vitro and in vivo on the NCL-H460 human NSCLC cell line. By using the co-delivery system, the overall effect was better than that of the single drug delivery NPs, with a higher cytotoxic effect, tumor-targeting capacity, and anti-tumor activity [127]. Another study proved that through co-encapsulation of cisplatin and paclitaxel in a single nano-formulation, with poly (lactic acid-co-glycolic acid)-poly (ethylene glycol) (PLGA-PEG) NPs, the efficiency of chemoradiotherapy is improved in mice with NSCLC. PLGA-PEG NPs improve the solubility and the pharmacokinetic profile of a wide range of small-molecule drugs, allowing the delivery of precise ratios of drugs, inducing combination synergy, and overcoming multidrug resistance mechanisms [128].

Chitosan-coated osimertinib-loaded biodegradable polymeric NPs targeting the EGFR T790M NSCLC mutant form were proven to have a 2.6- and 2.4-fold superior activity compared to plain osimertinib in H1975 mice models (cell line with epithelial morphology, isolated in 1988 from the lungs of a non-smoking female with NSCLC). Superior drug accumulation of 81.59 ± 5.8% was also observed in the novo NPs, with a higher triggered G2/M phase arrest. In vivo, the effects consisted of a reduction in tumor size and cytotoxicity [129].

Another study focused on the administration of erlotinib in resistant EGFR-mutated NSCLC and used NPs such as poly (ethylene glycol)-poly (lactic acid) (PEG-PLA NPs) for the co-delivery of erlotinib and fedratinib. The delivery system was more stable and could deliver the EGFR-targeted therapy more efficiently in acidic tumorous conditions. Fedratinib proved to be an important factor in the reversal of erlotinib resistance by downregulating the expression levels of proteins in the JAK2/STAT3 signaling pathway, including p-EGFR, p-JAK2, p-STAT3, and survivin. PEG-PLA NPs also had, both in vitro and in vivo, lower systemic side effects and a more potent effect on tumoral growth [130].

To combat resistance to EGFR inhibitors in NSCLC, He et al. [131] co-encapsulated EGFR and integrin αvβ3 inhibitors, namely erlotinib and cilengitide, respectively, in MPEG-PLA [methoxy poly (ethylene glycol)-poly (lactide)] NPs. The new molecule enhanced tumor suppression through cytotoxicity, with reduced organ damage, and reversed the drug resistance induced by integrin αvβ3.

When conjugating a biocompatible and photothermally conductive polymer (polypyrrole) to a TKI (afatinib) to direct near-infrared-photothermal therapy (NIR-PTT) in NSCLC, a research team found that polypyrrole-iron oxide-afatinib nanocomposite (PIA-NC) caused cancer cells to produce more reactive oxygen species (ROS), increased cytotoxicity, and minimized off-target biological effects of NIR-PTT [132]. 

Mutations located in the EGFR tyrosine kinase region (exon 19 deletion or exon 21 L858R mutation) are linked to patients’ positive response to gefitinib in NSCLC. Unfortunately, most patients developed acquired resistance and in over 60% of patients, the mechanism was the T790M EGFR mutation. By using targeted co-delivery of gefitinib and Rapamycin via aptamer-modified NPs, a research group induced cell autophagy. The explanation would be that by delivering Rapamycin in H1975 EGFR-mutant NSCLC cells, the EGF secretion increases, with a more potent effect of gefitinib on preventing cell proliferation [133].

#### 6.1.2. Lipid-Based Particles

Erlotinib (ETB), via oral administration, is used as the second-line therapeutic option for the treatment of metastatic NSCLC. Therefore, the local delivery at the tumoral site may affect the overall therapeutic impact. Bakhtiary et al. [134] combined ETB with solid lipid NPs (SLNs) as a dry powder inhaler. They tested the product on NSCLC human alveolar ADs epithelial A549 cells, the outcome being suitable flowability and aerodynamic traits and enhanced cytotoxic activity. By double inhibition of nuclear EGFR and PI3K/AKT, NPs that co-encapsulated erlotinib and quercetin had a more synergistic effect against A549 and NCI H460 cells than erlotinib with fisetin/carnosic acid/luteolin, with the reduction in expression of nuclear EGFR and an increased uptake in lung tissue. These types of NPs (EQNPs) have a small particle size of 87.3 ± 0.78 nm and release the highest quantity of erlotinib and quercetin at a pH of 5.5 [135].

Docetaxel (DTX) is an anti-neoplastic agent used in the treatment of advanced or metastatic NSCLC. Resveratrol (RSV) is a polyphenol, an anti-tumor agent, with the ability to inhibit the initiation, promotion, and progression stages of carcinogenesis and to enhance ROS production in cancer cells resulting in cytotoxicity. Specific lipid-based NPs have been created to deliver DTX and RSV in the mitochondria of EGFR-expressing tumor cells to overcome multi-drug resistance. In vitro and in vivo studies showed significant synergistic effects when using EGFR DTX/RSV LPNs, higher tumor inhibition ability, and the lowest systemic toxicity [136].

In order to overcome TKIs’ resistance in NSCLC, Yang et al. [137] created a poly (lactic-co-glycolic acid) porous microsphere dry powder that co-delivers afatinib and paclitaxel loaded in stearic acid-based solid lipid NPs and administered them via inhalation. Cell experiments showed synergistic effects of afatinib and paclitaxel and the experiments on mice indicated 96 h of high lung concentration with minimum adverse reactions but low concentrations in other tissues [137].

### 6.2. Magnetic Nanomaterials

Through combining anti-EGFR antibody (Cetuximab), as a molecular therapeutic, with hybrid plasmonic magnetic NPs, Yokoyama et al. [138] observed that EGFR-targeted C225-NPs are selectively taken up by EGFR-expressing NSCLC cells and had synergistic antitumor properties, also inducing apoptosis and autophagy. A more recent study from 2014 presented additional findings regarding the 225-NP treatment of EGFR-positive lung cancer, indicating that tumoral cells are being arrested in the G2/M phase of the cell cycle and suffer DNA damage, leading to effective tumor growth inhibition both in vitro and in vivo [139].

### 6.3. Inorganic Nanomaterials

Inorganic NPs lower the drug dose, prolong the retention time, and achieve targeted delivery, leading to increased cure rate and fewer complications. Moreover, they change the immunosuppressive environment and thus effectively deliver and extensively accumulate EGFR-TKIs in tumor site, reducing the accumulation of drugs in normal tissues [140].

Gold NPs (AuNPs) are used for the delivery of a variety of therapies in different types of cancer, including NSCLC. An example would be the C225-AuNPs novel compound, which contains a monoclonal antibody, Cetuximab (C225), targeting the external domain of EGFR, and the 14 nm gold NP as a carrier of C225. Both in vitro and in vivo, the results indicated a higher cytotoxicity and antitumor capability in A549 line NSCLC. C225-AuNPs also showed an increased suppression of the EGFR signaling pathway, probably via inducing membranous EGFR endocytosis and cytoplasmic EGFR accumulation [141]. Anti-EGFR peptide-conjugated PEGylated triangular gold nanoplates (TGN-PEG-P75) were used as targeting photothermal therapy (PTT) agents to treat NSCLC in mice, under the guidance of computed tomography (CT) and photoacoustic imaging. The TGN-PEG-P75 had uniform edge length (77.9 ± 7.0 nm), a neutrally charged surface, and a high affinity to EGFR-expressing cells via P75, with subsequently increased accumulation at the tumor site. TGN-PEG-P75 exhibited 3.8-fold superior therapeutic efficacy than TGN-PEG, with an increased inhibition of tumoral growth using PTT [142].

Near-infrared (NIR) emitting Ag2S quantum dots (QDs), when combined with Cetuximab antibody and 5-fluorouracil (5FU), proved to have an important effect on suppressing autophagy, compared to the stimulating effect of 5FU alone, that leads to resistance to cell death. These results were tested on low (H1299) and high (A549) EGFR-overexpressing cell lines and the outcome indicated a higher efficiency on A549 cells due to the induction of apoptosis [143].

### 6.4. siRNA Delivery Systems

Mad2 is a mitotic checkpoint component and its abolition leads to cell death. An EGFR-targeted chitosan NP was created to silence the *Mad2* gene using small interfering RNAs (siRNAs) in patients with A549 cell line NSCLC (epithelial carcinoma derived from a 58-year-old male patient, known to be KRAS mutant and EGFR wild type). chitosan is a positively charged biodegradable polymer and its main role is to protect the siRNAs from enzymatic activity, its efficiency being linked directly to its molecular weight. Higher molecular weight indicated a better outcome in vitro. The results showed a high tumoral uptake of the drug and a massive cell death by apoptosis [144]. EGFR-targeted chitosan NPs showed, in a more recent study, a consistent and preferential tumor targeting ability with rapid plasma clearance and the presence within the tumor up to 96 h. They exhibit a sixfold higher tumor targeting efficiency compared to the nontargeted NPs [145]. In a study from 2016, the efficiency of treatment was tested in lung cancer models (A549), both sensitive and resistant to cisplatin, as a single therapy or in combination with cisplatin. As a result, the siRNA-mediated Mad2 downregulation increased the sensitivity of lung cancer cells to cisplatin with the reversal of drug resistance and with the usage of lower doses to also decrease the adverse reactions. These outcomes were more significant in the targeted delivery group [146].

Hexagonal selenium NPs (HSNs) modified by siRNA (HSNM-siRNA) were used in a study in 2016 to target EGFR in human NSCLC and down-regulate the signaling cascade. This was assessed via Western blot and real-time PCR. The percentage of apoptotic cells and cell cycle progression were also measured after exposure to HSNM-siRNA and HSNs. The cell lines treated with HSNM-siRNA had a higher percentage of apoptotic cells and of cells in G1/G0 phase and a significantly decreased proportion of cells in S phase [147].

EGFR-mutation-positive NSCLC are usually treated with TKIs. Unfortunately, due to the development of resistance to this treatment, the drug efficacy is weakened. A multi-functional drug delivery system AP/ES was developed by using anti-EGFR aptamer (Apt)-modified polyamidoamine to co-deliver erlotinib and survivin-short hairpin RNA-expressing plasmid (shRNA). Survivin is an inhibitor of the apoptosis protein family present in many types of cancer cells and involved as a resistance factor in drug-induced apoptosis in NSCLC cells. In combination with Chloroquine, the AP/ES system overcame the drug resistance, both in vivo and in vitro, by normalizing tumor vessels for sufficient drug/gene delivery in Erlotinib-resistant NSCLC [148].

Radiation sensitizers, such as ChT, oxygen mimics, or metallic NPs in combination with ionizing radiation, are used increasingly more in NSCLC to improve the outcome for those receiving radiation therapy. A study by Reda et al. in 2019 [149] focused on Cetuximab conjugated NP that targets EGFR and delivers siRNA against polo-like kinase 1 (PLK1) (C-siPLK1-NP). The result indicated a downregulation of PLK1 expression and a G2/M arrest followed by cell death. In vivo, on A549 lung cancer cells, the combination of IR and C-siPLK1-NP resulted in immediate tumor control with eventual regression [149].

An in vitro study conducted by Majumder et al. [150] created a multicomponent and multifunctional cancer-targeted delivery system, containing Nanostructured Lipid Carriers (NLCs) as vehicles, luteinizing hormone-releasing hormone (LHRH) as a cancer-targeting moiety, siRNA targeted to EGFR mRNA as a suppressor of EGF receptors, EFG-TK inhibitor gefitinib and/or paclitaxel as anticancer drugs, and an imaging agent (Rhodamine) for the visualization of cancer cells. The drug entrapment efficiency of gefitinib and paclitaxel was greater than 90% (90.54 ± 5.48% and 97.60 ± 0.34%, respectively). Both the gefitinib and paclitaxel-loaded NLCs showed 5 to 10-fold improved in vitro anticancer activity in a series of human lung cancer cells when compared with their parent drugs. They proved the superiority of using a single drug with multiple components rather than delivering them separately, with the possibility of detection of drug-resistant NSCLC, higher efficiency of treatment, and fewer adverse reactions [150]. Polyethylenimine (PEI) lipid NPs in combination with siRNA complex (EPV–PEI–LNP–siRNA) were used to target PD-L1 and EGFR in NSCLC. The siRNA and EGFR short peptide vaccine had a high biocompatibility, showed effective tumor immunotherapy, and had an effective role in the downregulation of the expression of PD-L1 in cells compared to the blank group and the PD-L1-siRNA group [151].

The usage of edible and cation-free kiwi-derived extracellular vesicles (KEVs) loaded with Signal Transducer and Activator of Transcription 3 interfering RNA (siSTAT3), with a size of 186 nm, exhibited high stability, specificity, and cytotoxicity in vivo in EGFR over-expressing and mutant PC9-GR4-AZD1 cells (lung AD cell line with deletion in exon 19 of the *EGFR* gene and high sensitivity to TKIs). In mice, the systemic delivery of STAT3/EKEVs suppressed tumor xenografts via STAT3-induced apoptosis, combating the EGFR resistance [152]. 

### 6.5. Mesoporous Silica Nanomaterials

Cetuximab-capped mesoporous silica NP (MP-SiO_2_ NP) loaded with gefitinib, proved to be of higher efficiency in inhibition of cell growth, in EGFR-mutant NSCLC with gefitinib-resistant cell line derived from PC9 cell (PC9-DR), than gefitinib alone. By using cetuximab-capped MP-SiO_2_ NP as a drug carrier, gefitinib entered cells in a greater quantity through endocytosis and the high glutathione levels increased its local effect and overcame TKIs resistance [153].

### 6.6. NUFS Nanomaterials

One of the major complications of NSCLC is the presence of metastases in the central nervous system, with low penetration ability of the TKIs via the blood–brain barrier. A study conducted by Kim et al. [154] stated the efficacy of water-soluble erlotinib (NUFS-sErt) against these metastases. There was no difference between the new agent and erlotinib alone in terms of inhibiting the proliferation of cancer cells and suppressing EGFR signaling in vitro and in vivo but by injecting NUFS-sErt into the brain ventricle, a significant tumor growth inhibition was observed in an intracranial xenograft model, indicating a possible alternative treatment for patients with central nervous system metastases [154].

## 7. Discussion

Nanotherapy is an important tool in improving lung cancer management and has great potential for future personalized treatments of various cancers, but it still needs optimization. The barrier to widespread clinical use of targeted NPs is not technical in nature, as indicated by the wide variety of drug-containing targeted NPs produced by researchers throughout the world, many of which have been presented in this review. A synopsis of the characteristics and mechanisms of EGFR-targeted NPs as well as their weaknesses and strengths are depicted in Table 2.

Practical improvement is necessary for polymeric NPs since difficult IV use with low solubility has been reported [121] as well as concerns with particle stability having been described [123]. As with every drug, reservations exist concerning the uncontrollable accumulation of NPs in healthy tissues, leading to systemic cytotoxic effects [125,126,127]. The liver and kidneys seem to be especially affected, as shown by Tian et al. [128], who analyzed PLGA-PEG NPs delivering PTX and fatty acid-modified CP prodrug (CPP) to lung cancer cell lines [155], founding miscellaneous NPs biodistributed to the tumor target as well as the liver and the spleen. In addition, around 20–30% of NSCLC with an activating mutation display an intrinsic resistance to EGFR-TKI, the fast mutational characteristics leading to 50% resistance to first- and second-generation EGFR-TKIs within 9 to 14 months. To improve the EGFR inhibition, targeting several parts within the EGFR cascade or several parallel pathways to prevent cross-activation of the EGFR have been considered [156,157]. 

It was demonstrated that drug-laden targeted NPs can be produced reliably, at a large scale, and in accordance with pharmaceutical good manufacturing practice (GMP) principles [150,151], the production processes being adequate for testing targeted NPs in human patients in multiple clinical trials.

A review of nine clinical trials of non-targeted NPs in the treatment of lung cancer (seven using paclitaxel albumin-stabilized NPs, one using CRLX101, and one using ABI-009) concluded that these treatments were not inferior to the standard of care, with widely varying rates of serious adverse events [158]. No clinical trials of EGFR-targeted NPs in lung cancer have been identified to date but three trials using anti-EGFR immunoliposomes containing Doxorubicin (anti-EGFR ILs-dox) were conducted for the treatment of other types of cancer [159]. A phase I dose escalation study (clinical trial identifier NCT01702129) in patients with pancreatic, head and neck, colorectal, and urothelial cancers concluded that anti-EGFR ILs-dox were well tolerated and therefore warranted further use in phase II trials [160]. A phase II trial of anti-EGFR ILs-dox in glioblastoma (NCT03603379) concluded that the therapy was safe but that NPs could not cross the intact blood–brain barrier to treat central nervous system tumors [161]. Another phase II trial of anti-EGFR ILs-dox in advanced triple-negative breast cancer (NCT02833766) did not meet its primary endpoint of progression-free survival at 12 months [162].

Overall, the results of EGFR-targeted NPs in clinical trials have been underwhelming. This could be due to either insufficient understanding of NP behavior in the human body and their uptake by tumor cells or it could be due to insufficiently advanced NPs designs. A recent review by Fan et al. [163] described NPs targeting efforts on three levels: tissular (reaching the tumor inside the body), cellular (reaching the cancer cells once in their vicinity), and sub-cellular (unloading the therapeutic agents in the right organelles for maximum effect) [163]. 

The preparations tested in clinical trials lack the complete triple-tiered designs aimed at achieving the desired NP localization at every level, but, on the bright side, safety and tolerability were acceptable in many targeted NP clinical trials. 

Perhaps the next steps on this road should include combining tissular, cellular, and sub-cellular targeting mechanisms into a single therapy to achieve maximum effect. 

## 8. Conclusions

Targeted EGFR nanotherapy offers a promising approach to NSCLC management in the context where traditional ChT has limited efficacy in treating NSCLC and often causes significant side effects by its non-specific nature. Preclinical studies demonstrated that targeted delivery of ChT drugs or EGFR-TKIs to NSCLC using EGFR–antibody conjugated NPs can enhance cytotoxicity and reduce off-target toxicity compared to free drugs. Combination NPs co-encapsulating EGFR-TKIs with ChT drugs or targeted agents showed synergistic effects in vitro and in vivo in overcoming resistance; also, EGFR-targeted NPs delivering siRNA against resistant genes helped reverse drug resistance. 

These promising preclinical findings suggest that EGFR-targeted nanotherapy has the potential to improve NSCLC treatment through enhanced tumor targeting, cytotoxicity, and overcoming resistance. However, further optimization of NP delivery systems and comprehensive evaluation in clinical trials are needed before translation to patients. Future research should focus on improving tumor specificity, drug loading, release kinetics, and the stability of NPs while demonstrating safety and efficacy in vivo. Overall, EGFR-targeted nanotherapy is a promising novel strategy warranting further development as a personalized therapy for NSCLC patients with EGFR mutations. 

## Figures and Tables

**Table 2:** Synthetic table of specific EGFR-targeted nanoparticles.

Ref.	Study Type	Composition	Drug Delivered	Size	Surface Mods	Mechanism	Weaknesses	Strengths
[123]	In Vivo Study	Polymer NPs	Heparin-Cisplatin	20 nm	Negative charge, hydrophilic	EGFR targeting	Particle stability, organ conc., side effects	Enhanced antitumor effect, reduced toxicity
[125]	In Vitro and In Vivo	PLA NPs	Gemcitabine − Cetuximab	120 nm	EDC activation	EGFR signal block	Cytotoxic to normal cells	Enhanced cell killing, passive targeting
[126]	In Vitro Study	Chitosan NPs	Lipid-Modified Cisplatin	220–365 nm	Positively charged	Receptor-mediated endocytosis	Side effects	Improved cytotoxicity
[127]	In Vitro and In Vivo	PLGA-PEG NPs	Paclitaxel + Carboplatin	125 nm	Negative charge	Drug release	Systemic side effects	Sustained drug release
[128]	In Vitro and In Vivo	PLGA-PEG NPs	PTX + Fatty Acid CPP	80–85.5 nm	Negative charge	NP phagocytosis	Biodistribution, liver and kidney	Enhanced apoptosis
[129]	In Vitro and In Vivo	Chitosan-coated NPs	Osimertinib	101.3–119.7 nm	Biodegradable NPs	Drug release	Side effects	Reduced tumor size
[130]	In Vitro and In Vivo	PEG-PLA NPs	Erlotinib + Fedratinib	120 nm	Hydrophobic, dual-drug	Acidic microenvironment	Side effects	Enhanced therapeutic efficacy
[131]	In Vitro and In Vivo	Chitosan NPs	Osimertinib	101.3–119.7 nm	Biodegradable NPs	Drug release	Side effects	Reduced tumor size
[134]	In Vitro	Solid Lipid NPs	Erlotinib microparticles	1–5 μm	Dry powder inhaler	PI3K/AKT signaling	Inhalatory admin.	Suitable flowability
[135]	In Vitro and In Vivo	Polymer NPs	Erlotinib + Quercetin	87.3 ± 0.78 nm	Chitosan-MA-TPGS	Nuclear EGFR	Low side effects	Minimal injury to healthy tissue
[136]	In Vitro and In Vivo	Core-Shell Lipid-Polymer NPs	Docetaxel + Resveratrol	189.6 ± 5.6 nm	Dual-drug loaded NPS	Mitochondrial targeting	Mouse weight loss	Higher tumor inhibition
[137]	In Vitro and In Vivo	Solid Lipid NPs	Afatinib + Paclitaxel	500 nm	Dual-drug loaded NPS	PI3K/Akt/mTOR pathway	Hepatic edema	Increased cell migration inhibition
[138]	In Vitro	Magnetic NPs	C225 + Hybrid Plasmonic NPs	54 ± 11 nm	Gold-coated iron oxide NPs	Apoptosis, autophagy	Multivalency effect	Higher efficiency
[139]	In Vitro and In Vivo	Magnetic NPs	C225 + Hybrid Plasmonic NPs	73 ± 35 nm	Gold-coated iron oxide NPs	Autophagy, apoptosis	Active on EGFR-positive cells	Greater tumor suppression
[141]	In Vitro and In Vivo	Gold NPs	Cetuximab	25 nm	BSA-treated	EGFR endocytosis	Time/dose-dependent effect	Increased cytotoxicity
[142]	In Vitro and In Vivo	Gold Nanoplates	Anti-EGFR PTT agent	77.9 ± 7.0 nm	Neutrally	Photothermal therapy	Requires light exposure to activate the photothermal effect	Selectively kill cancer cells, minimal side effects, can be used for imaging
[143]	In Vitro and In Vivo	Ag2S QDs	Cetuximab functionalization	<50 nm	PEGylated cationic NPs	Endocytosis	Fluorescence imaging	Enhanced apoptosis
[144]	In Vitro	PEG-CS NPs	Mad2 siRNA	100–250 nm	Peptide-modified PEG-CS NPs	EGFR internalization	Efficiency dependent on MW	Increased selectivity
[145]	In Vitro and In Vivo	NTG and TG CS NPs	Mad 2 siRNA	113.1–230.1 nm	Peptide-modified PEG-CS NPs	Apoptosis	Organ accumulation	Higher targeting efficiency
[146]	In Vitro and In Vivo	NTG and TG CS NPs	Mad 2 siRNA + Cisplatin	126.7–202.7 nm	PEGylated CS derivatives	Apoptosis, mitotic failure	Decreased plasma exposure	Minimized side effects
[147]	In Vitro	Hexagonal Selenium NPs	siRNA	20 nm	Oligonucleotide modification	Down-regulation of *EGFR* genes	Increased apoptosis, suppression	Effective tumor immunotherapy
[150]	In Vitro and In Vivo	Nanostructured Lipid Carriers	Gefitinib + Paclitaxel + siRNA	100–300 nm	LHRH-coated NLCs	Suppression of EGF pathway	Instability of siRNA	Enhanced internalization
[151]	In Vitro and In Vivo	PEI Lipid NPs + siRNA	EGFR + PD-L1 siRNA	30 nm	Peptide-modified PEI	Immune stimulation	T cells-related adverse effects	High biocompatibility, tumor immunotherapy
[152]	In Vitro and In Vivo	Kiwi-Derived Extracellular Vesicles	siSTAT3	186 nm	Aptamer surface mod.	STAT3-induced apoptosis	Side effects	High specificity, cytotoxicity
